# Late-developing tongue adenoid cystic carcinoma after pulmonary metastasectomy: a case report

**DOI:** 10.1186/1477-7819-12-102

**Published:** 2014-04-21

**Authors:** Young Jo Sa, Sung Bo Sim, Tae-Jung Kim, Seok Whan Moon, Chan Beom Park

**Affiliations:** 1 Department of Thoracic and Cardiovascular Surgery, College of Medicine, The Catholic University of Korea, 222 Banpo-daero, Seocho-gu, Seoul 137-701, Republic of Korea; 2 Department of Hospital Pathology, College of Medicine, The Catholic University of Korea, 222 Banpo-daero, Seocho-gu, Seoul 137-701, Republic of Korea

**Keywords:** Lung cancer surgery, Metastasectomy, Adenoid cystic carcinoma

## Abstract

Adenoid cystic carcinoma (ACC) is a relatively rare epithelial tumor of the salivary glands that accounts for approximately 5 to 10% of all salivary gland neoplasms. The typical clinical and pathological findings of this tumor include slow indolent growth, common local recurrence, and late distant metastasis to lung, brain, bone, liver, thyroid, and spleen. We report a 52-year-old female patient who presented a tongue ACC, 27 months after successful pulmonary ACC resection.

## Background

Adenoid cystic carcinoma (ACC) is a malignant neoplasm that usually arises in the salivary glands [1], but can also occur in the breast, skin, uterine cervix, upper digestive tract, and lung [2]. Primary pulmonary ACC accounts for less than 0.2% of all lung cancers, and only 10% of all primary pulmonary ACCs are peripheral in origin [3]. To the best of our knowledge, peripheral pulmonary ACC detected prior to its appearance in the tongue has not been reported previously in the literature. We report herein a case of peripheral ACC in the right lower lobe, which was followed 27 months later by tongue ACC.

## Case presentation

**Figure 1:**
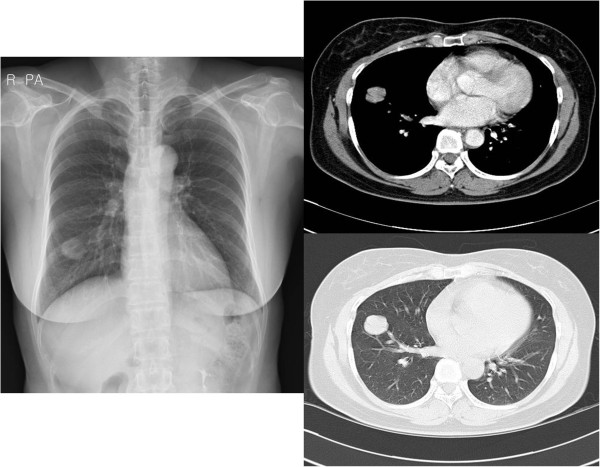
**Chest X-ray shows a solitary mass in the right lower lobe of the lung.** Chest CT shows a relatively well-circumscribed and enhanced mass arising from bronchi smaller than the segmental bronchus. CT, computed tomography.

**Figure 2:**
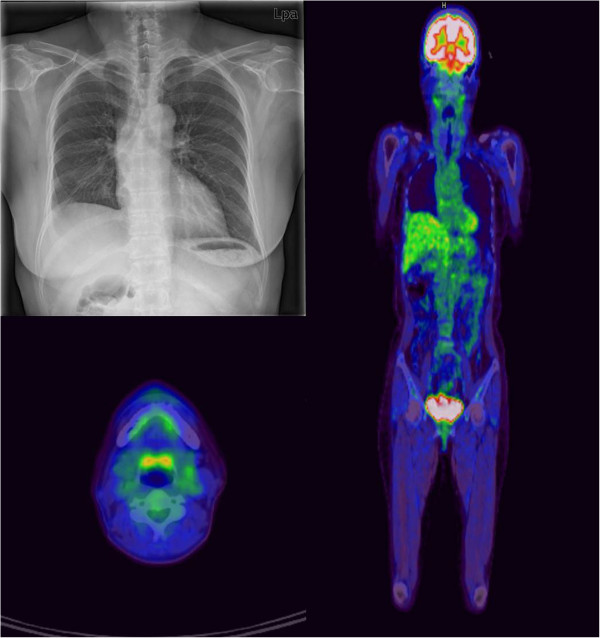
**Chest X-ray and PET-CT after lobectomy show that the tumor from the salivary glands could not be detected clinically.** The diagnosis made at that time was thus primary peripheral pulmonary ACC. ACC, adenoid cystic carcinoma; CT, computed tomography; PET, positron emission tomography.

A 52-year-old woman was referred to Yeouido St. Mary’s Hospital, Republic of Korea for evaluation of an incidentally found right lower lobe lung mass. The patient had a history of hypertension and denied recent weight loss or smoking. The patient’s initial laboratory findings were unremarkable, and the results of a pulmonary function test were normal. A chest computed tomography (CT) scan revealed a relatively well-circumscribed and enhanced mass involving the visceral pleura in the right lower lobe, and no mediastinal lymphadenopathy (Figure 1). The mass was biopsied under video-assisted thoracoscopic surgery, and an intraoperative pathologic diagnosis revealed malignant lung cancer. The patient underwent right lower lobectomy and mediastinal lymph node dissection. A histopathological examination disclosed ACC with a cribriform and tubular pattern; metastasis was not found in the dissected lymph nodes. For confirmation of a primary or metastatic tumor, an otolaryngologic examination and whole-body positron emission tomography (PET) were performed after lobectomy (Figure 2). The patient was diagnosed with primary peripheral pulmonary ACC because of no evidence of salivary gland tumor or other metastasis; neither radiotherapy nor chemotherapy was performed after lobectomy.

**Figure 3:**
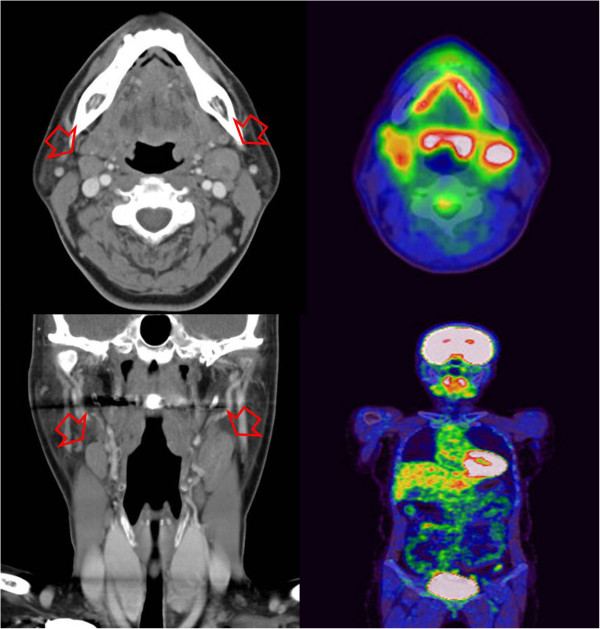
**Neck CT and whole-body PET-CT obtained 27 months after lobectomy.** The neck CT shows that the lymph nodes on both sides of the neck are enlarged (arrows), and the PET-CT discloses increased 18F-FDG uptake in the mid-portion of the tongue base, and enlarged lymph nodes. 18F-FDG, 18F-fluorodeoxyglucose; CT, computed tomography; PET, positron emission tomography.

**Figure 4:**
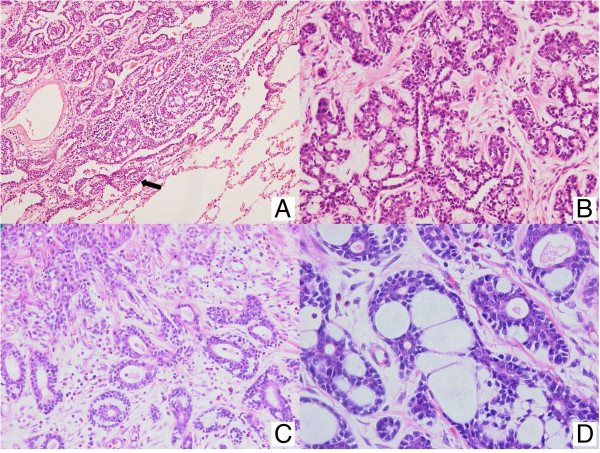
**Microscopic findings of the lung and tongue tumor specimen. (A,B)** A lining of tumor cells can be seen along the alveolar walls (arrow) (H &; E, x100). Higher magnification view of the tumor cells shows a predominantly cribriform or tubular growth pattern with numerous gland-like spaces containing homogenous acidophilic materials. **(C,D)** Squamous cell epithelium and ACC of the tongue (H &; E, x200). Microscopic findings show a predominantly cribriform or tubular growth pattern with numerous gland-like spaces containing mucopolysaccharide, consistent with ACC (H &; E, x400). ACC, adenoid cystic carcinoma; H &; E, hematoxylin and eosin.

During follow-up, the patient was diagnosed and treated for transitional cell cancer of the bladder. Recurrence of the bladder cancer was suspected at 27 months after the pulmonary operation, and a whole-body PET scan showed an enlarged and increased uptake pattern of both parotid nodules without recurrence in the lung (Figure 3). The bladder nodule was benign and the parotid nodules disclosed ACC of the tongue with metastasis to the parotid lymph node. The otolaryngologist performed wide excision of the tongue cancer. The pathologic diagnosis was ACC of the tongue; identical to those of the resected right lower lobe lung mass taken 27 months previously (Figure 4). After 5 weeks of adjuvant chemoradiation therapy, the patient remained in good health and with no detectable recurrence at 7 months of follow-up.

## Discussion

We represent a case with the following unusual manifestations of ACC: 1) the initial presentation was a solitary peripheral lung mass; 2) the primary site of ACC was the tongue; and 3) the primary lesion was found 27 months after successful treatment of the metastatic lesion.

ACC constitutes approximately 5 to 10% of all salivary gland tumors. It is particularly prevalent in the palate, but also in the submandibular and parotid glands, and the tongue [4]. Although the optimal management of this unusual disease is surgical resection, ACC of salivary gland origin has a high incidence of locoregional recurrence and distant metastasis [2,5].

In the lung, salivary gland-type tumors can occur as either primary tumors or metastatic tumors arising in the salivary glands. Most primary pulmonary ACCs arise in the submucosal glands of the trachea and main bronchus, and approximately 10% of ACCs are located peripherally. Multiple lesions usually indicate a pulmonary metastasis, while a solitary lesion may be an indication for metastasectomy in ACC of the salivary gland. Distinguishing primary from metastatic lung ACC based on histology is difficult [6], and history taking and clinical examination are important.

Tongue ACCs are rarely reported [4], and a recent study [7] found that the overall 5- and 10-year survival rates of tongue ACCs were 51% and 34%, respectively. Pulmonary metastases develop in 37% of tongue ACCs. Surgery is an important treatment option, but the use of adjuvant chemotherapy or radiotherapy remains controversial.

While we could not confirm by pathologic examination whether the lung lesion was a primary tumor or a metastasis, our patient was diagnosed with a primary peripheral pulmonary ACC after the initial surgery because no evidence of a tumor origin was found on whole-body PET scan and the otolaryngologic examination. We were surprised to observe the pathologic findings of tongue ACC, the cribriform and tubular pattern of which was identical to that observed in the specimen of pulmonary cancer removed 27 months earlier. There are three potential relationships between pulmonary ACC and tongue ACC: 1) subclinical tongue ACC with metastatic pulmonary ACC 27 months previously; 2) metastatic tongue ACC; and 3) primary pulmonary ACC followed by primary tongue ACC.

Metastatic ACC of the salivary gland or tongue has not been reported previously. However, slow indolent growth and late distant metastasis are common findings of ACC in the salivary gland, and pulmonary metastasis is also frequent in tongue ACC [7]. Identical pathologic findings and the same pattern of immunohistochemical staining suggest that this is unlikely to be a case of double primary ACC. Therefore, late presentation of subclinical tongue ACC after resection of metastatic pulmonary ACC was considered reasonable.

## Conclusions

We have presented herein a case of ACC with unusual clinical behaviors that remain to be fully defined. Although ACC is characteristically slow growing and associated with late distant metastasis, our case showed the reverse presentation pattern: early recognition of a metastasis and late presentation of the primary site. Therefore, peripheral pulmonary ACC should be carefully followed and considered in relation to head and neck ACC, even after successful management.

## Consent

Written informed consent was obtained from the patient for publication of this case report and any accompanying images. A copy of the written consent is available for review by the Editor-in-Chief of this journal.

## Abbreviations

18F-FDG: 18F-Fluorodeoxyglucose; ACC: Adenoid cystic carcinoma; CT: Computed tomography; H &; E: Hematoxylin and eosin; PET: Positron emission tomography.

## Competing interests

The authors declare that they have no competing interests.

## Authors’ contributions

YJS participated in the preparation of the manuscript, literature search, and drafted the manuscript. SBS provided assistance for the operation. SWM participated in the study design and coordination. TJK diagnosed the pathology of this case. CBP edited the manuscript for its scientific content and conceived of the study. All authors read and approved the final manuscript.
